# Hypermutation as an Evolutionary Mechanism for *Achromobacter xylosoxidans* in Cystic Fibrosis Lung Infection

**DOI:** 10.3390/pathogens9020072

**Published:** 2020-01-21

**Authors:** Laura Veschetti, Angela Sandri, Helle Krogh Johansen, Maria M. Lleò, Giovanni Malerba

**Affiliations:** 1 Laboratory of Computational Genomics, Department of Neurosciences, Biomedicine and Movement Sciences, University of Verona, 37134 Verona, Italy; laura.veschetti@univr.it (L.V.); giovanni.malerba@univr.it (G.M.); 2 Department of Diagnostics and Public Health, Microbiology Section, University of Verona, 37134 Verona, Italy; angela.sandri@univr.it; 3 Department of Clinical Microbiology, Rigshospitalet, 2100 Copenhagen, Denmark; hkj@biosustain.dtu.dk; 4 Department of Clinical Medicine, Faculty of Health and Medical Sciences, University of Copenhagen, 2200 Copenhagen, Denmark

**Keywords:** lung infection, opportunistic pathogen, bacterial evolution, comparative genomics, clonal diversification

## Abstract

*Achromobacter xylosoxidans* can cause chronic infections in the lungs of patients with cystic fibrosis (CF) by adapting to the specific environment. The study of longitudinal isolates allows to investigate its within-host evolution to unravel the adaptive mechanisms contributing to successful colonization. In this study, four clinical isolates longitudinally collected from two chronically infected patients underwent whole genome sequencing, de novo assembly and sequence analysis. Phenotypic assays were also performed. The isolates coming from one of the patients (patient A) presented a greater number of genetic variants, diverse integrative and conjugative elements, and different protease secretion. In the first of these isolates (strain A1), we also found a large deletion in the *mutS* gene, involved in DNA mismatch repair (MMR). In contrast, isolates from patient B showed a lower number of variants, only one integrative and mobilizable element, no phenotypic changes, and no mutations in the MMR system. These results suggest that in the two patients the establishment of a chronic infection was mediated by different adaptive mechanisms. While the strains isolated from patient B showed a longitudinal microevolution, strain A1 can be clearly classified as a hypermutator, confirming the occurrence and importance of this adaptive mechanism in *A. xylosoxidans* infection.

## 1. Introduction

Cystic fibrosis (CF) is strictly linked with chronic bacterial respiratory infections; in fact, airways infection and the ensuing inflammation account for the majority of morbidity and mortality of CF patients [1]. *Achromobacter xylosoxidans* is an opportunistic pathogen in patients with CF, where this microorganism can survive for a long time in both lower and upper airways [2]. Infection results either from acquisition of bacteria present in the environment or from direct or indirect transmission [3,4] and is usually complicated by the intrinsic and acquired multidrug resistance traits carried by this microorganism. Chronic lung infection by *A. xylosoxidans* has been associated with decline in respiratory function, increased frequency of exacerbations and lung inflammation [3,5,6]. Nevertheless, very little is known about the pathogenic mechanisms allowing *A. xylosoxidans* to colonize and persist in CF airways.

Pathogenic mechanisms of other CF pathogens are well-known; in particular, *Pseudomonas aeruginosa* has been extensively studied, due to its high incidence in CF patients and the difficult—in many cases impossible—eradication. One of the main mechanisms favouring the persistence of *P. aeruginosa* in the airways of CF patients is the ability to genetically adapt during chronic infection. In particular, short-term adjustments are believed to be the result of regulatory alterations in gene expression whereas long-term adaptation is the result of the accumulation of pathoadaptive mutations [7]. Consequently, the potential and speed for bacterial pathogens to genetically adapt to the host immune system and drug therapies may be determined by the within-host mutation rate [8]. Interestingly, the generation rate of mutations can be accelerated due to mutations in the DNA mismatch repair system, giving rise to hypermutation events and to clonal diversification within the host [9]. The adaptation of bacteria to a heterogeneous and changing environment can promote selection of hypermutable strains. Since *A. xylosoxidans* is an emerging pathogen, its pathoadaptive evolution and its impact in relation to chronic infections is still not clear. In this work we studied longitudinally collected clinical isolates of *A. xylosoxidans* to unravel the adaptive mechanisms contributing to its successful colonization of CF lungs.

## 2. Results

We sequenced the genome of four *A. xylosoxidans* isolates longitudinally collected from sputum samples of two CF patients. Two isolates were collected from each patient: in 2005 and 2008 from patient A, and in 2008 and 2014 from patient B. Genotypic relatedness was verified by checking core genome similarities: Isolates coming from each patient showed 87% similarity with the respective longitudinal isolate. Both patients had been chronically infected for 9 years: The year of first *A. xylosoxidans* isolation was 1996 and 1999 for patients A and B, respectively. The general information on de novo assembly of each genome is presented in Table 1. The assembly lengths varied between 6.6–6.9 Mbp and the GC-content varied between 67.63–68.09%, which is comparable to the published reference genome NH44784-1996 [10]. 

Sequence reads were aligned to the reference genome and to the de novo assembly of the other isolate from the same patient (i.e., reads from A1 isolate were aligned to the de novo assembly of A2 and vice versa) to investigate genetic changes over time. The mapping reads percentage is shown in Table 2. 

Since a high number of mapping reads was obtained between the de novo assemblies (range: 96.49–98.78%), this approach allowed us to detect almost all the variants between longitudinal isolates. Differently, carrying out the analysis by only mapping the reads against the reference genome, these variants would not have been considered, as the lower number of mapping reads suggests.

### 2.1. Variant Analysis

To take into account the possibility of calling false positive variants within the same patient over time, the sequencing reads of A1 and B1 isolates were mapped on the genome assembly of A2 and B2 respectively, and vice versa, and only the intersection of the two groups of variants was taken into consideration. A total of 187 variants was identified in patient A genomes, while a total of only eight was identified in genomes from patient B as shown in Table 3. The majority of genetic alterations were represented by SNPs. Moreover, the greater number of SNPs in patient A genomes (85%) is due to transitions and not transversions (mean ratio: 5.8), which is typical of hypermutators. 

Since a great number of variants were identified in strains from patient A, the variant analysis was performed also against the reference genome in order to investigate variants independently. A mean total of 116 variants were found in patient A while a mean total of only 26 were found in patient B. As shown in Table 5, the genome of isolate A1 harboured a greater number of variants (n = 162), particularly SNPs (n = 150), when compared to the other isolates; even twice as many SNPs than its longitudinal isolate A2. Moreover, 73% of A1 SNPs are due to transitions (transitions/transversions ratio: 2.75), supporting the possibility that a hypermutation event occurred.

As shown in Table 3, among the mutations that could cause a protein loss of function, frameshift mutations are the majority followed by stop gain mutations. In particular, genomes of patient A show a higher number of mutations when compared to genomes of patient B. A2 and B1 genomes do not harbour mutations with a predicted high impact on protein function, since the missense SNPs have all been annotated as having a moderate impact; the B2 genome harbours only three mutations, while frameshift mutations are the majority in the A1 genome. Gene products affected by mutations identified through comparison between longitudinal isolates are summarized in the Supplementary material (Table S1).

The list of genes presenting variants, divided by functional classes according to the protein function as reported on UniProt database, is shown in Table 4. In genomes from patient A, in particular in A1 genome, the majority of genes presenting variants are involved in metabolism (39%), followed by genes involved in transcription and translation (12%) and transporter proteins encoding genes (12%). 

### 2.2. Genetic Basis of Hypermutation

Since we suspected that a hypermutation event occurred in A1 isolate, the occurrence of genes involved in DNA repair was investigated in all four genomes. All clinical isolates differed from the reference genome because they harboured two copies of the *mutL* gene; they lacked superoxide dismutase genes *sodA, sodB, sodC* and DNA repair gene *radC*; and they had only one copy of nucleotide excision repair genes *uvrA* and *uvrB*. Moreover, the isolates coming from patient B presented an extra copy of *uvrD*. Genes involved in DNA repair in each clinical isolate and in the reference strain are summarized in the Supplementary material (Table S2).

Furthermore, gene sequences were thoroughly examined and a 95 nucleotides deletion that translated to a 36 amino acids gap was found in A1 *mutS* gene (reference locus tag NH44784_RS30630). No other mutations in DNA repair genes were found in any other genome. The mutation of *mutS* might explain the higher rate of SNPs found in A1, thus defining this isolate as a hypermutator.

### 2.3. Mobile Genetic Elements

The presence of mobile genetic elements such as phages, plasmids, integrative and conjugative elements (ICEs), integrative and mobilizable elements (IMEs), and *cis*-mobilizable elements (CIMEs), was investigated. For phages research, only complete regions were considered. As shown in Table 5, *Burkholderia* sp. phages (KS9, Bcep176, BcepMu, KS14) were identified in all genomes. In addition, A2 also carries phages 118970 and YMC11/02/R656 from *Salmonella* sp. and *Pseudomonas* sp. respectively.

ICEs were detected in isolates from patient A. In A1, we found one putative ICE containing genes related to Type 4 Secretion System (T4SS), bicyclomycin resistance and iron metabolism. A2 presented one putative ICE-containing gene related to T3SS, multidrug resistance, motility, virulence and metabolism. Both B1 and B2 presented the same putative IME-containing genes related to multidrug resistance.

Although plasmids were not found, the presence and variation of phages, ICEs and IMEs illustrates the potential ability of *A. xylosoxidans* to carry genetic and transferable elements that could contribute to the dissemination/acquisition of microbial functions.

### 2.4. Phenotypic Features

**Figure 1:**
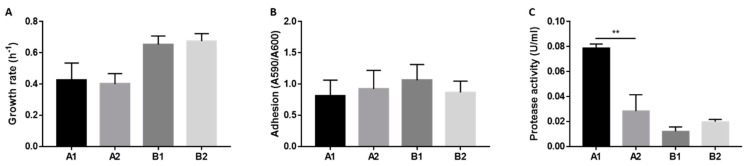
Growth rate (**A**), biofilm formation (**B**) and protease activity (**C**) of *A. xylosoxidans* isolates. Growth rate was calculated from 24 h growth curves in LB medium (**A**). Adhesion was measured by crystal violet staining of surface-attached bacteria divided by A_600_ of non-attached bacteria (B). Protease activity was measured in culture supernatant by azocasein assay. Protease activity is expressed as enzymatic units per ml (C). Each value represents the mean ± SEM of three experiments. Statistical analysis was performed by t test, ** *p* < 0.01.

To investigate possible phenotypic variations within the same host over time that might be related to hypermutation, we evaluated features such as growth, virulence and antibiotic resistance, which are known to undergo modifications during bacterial adaptation. As shown in Figure 1, no significant changes in terms of growth rate and adhesion ability occurred in the isolates from the two patients. However, we measured a strong protease activity in the culture supernatant of A1 isolate, that was significantly higher in comparison to strain A2. No such variation was observed between the isolates from patient B. As concerns antibiotic susceptibility, only A1 strain showed increased resistance to meropenem (MIC > 8 mg/L) [11].

## 3. Discussion

By sequencing and analysing the genomes of longitudinal isolates of *A. xylosoxidans* collected from two infected CF patients, we have identified different adaptive mechanisms of *A. xylosoxidans* to survive in a hostile environment like the CF lungs. 

Two isolates were longitudinally collected from each patient with an interval of 3 years in patient A and 6 years in patient B. During this time, both patients were chronically infected. In addition to the standard comparison with a reference strain, we compared each *A. xylosoxidans* genome with its longitudinal isolate in order to study genomic variation avoiding biases linked to the choice of the reference genome. In all genomes, the majority of genes presenting variants are involved in metabolism, as previously shown in other studies [7], followed by genes involved in transcription and translation and transporter proteins encoding genes. Metabolic regulation, gene expression and trade-off are evidently major targets of adaptation, probably enabling a physiological adaptive response through changes in regulatory genes [12,13].

When mutations accumulate at a high rate throughout the genome, we are usually in the presence of so-called hypermutators. Hypermutation arises through mutations that disrupt the methyl-directed mismatch repair (MMR) system, and *mutS* inactivation is the most widespread defect [14]. Although a previous study reported the finding of possible *A. xylosoxidans* hypermutable isolates in a CF patient [7], this conclusion was weakened by the absence of mutations in the MMR genes. On the contrary, in this study we identified an isolate (namely A1) that not only presents a high number of SNPs mainly due to transitions but also carries a large deletion in *mutS* gene, thus clearly defining this isolate as a hypermutator. 

While this suggests the co-evolution of sub-populations from an original infecting strain in patient A, isolates of patient B seem to represent a situation of longitudinal microevolution. Both evolutionary mechanisms—hypermutation and microevolution—enabled long-term adaption in CF lungs, although it has been previously suggested that high mutation rates offer bacterial advantages in pathogenesis. In fact, hypermutation has been credited with facilitating the phenotypic changes and clonal diversification characteristic of *P. aeruginosa* adaptation to the CF lung environment [15,16]. Furthermore, MMR-deficient hypermutators are overrepresented in populations of various other pathogenic bacteria such as *Escherichia coli, Salmonella* spp., *P. aeruginosa* and *Staphylococcus aureus* [17]. Populations that are rapidly adapting to new or changing environments usually provide opportunities for hypermutable genotypes to rapidly spread beneficial mutations. However, the emergence of hypermutators does not always accelerate adaptive evolution. It has also been shown that hypermutator populations do not always produce greater fitness gains than DNA-repair proficient populations [18]. A mutator might rise transiently to high frequency and then be eliminated if the non-mutator type produces an even more beneficial mutation than that produced by the mutator [19]. For instance, the high protease secretion as well as meropenem resistance observed in the hypermutator isolate A1 could have been a result of adaptation to a stressful condition, but probably did not produce a greater fitness. Indeed, non-mutator strain A2 was isolated 3 years later, suggesting that even a low mutation rate was sufficient to generate beneficial mutations, similarly to the microevolution observed in patient B.

In conclusion, we report the occurrence of hypermutation as a mechanism that could be involved in *A. xylosoxidans* long-term infection of CF lungs. Further studies, possibly on a larger scale, will be needed to understand the frequency and benefits of *A. xylosoxidans* hypermutability.

## 4. Materials and Methods

### 4.1. Bacterial Isolates

Four clinical isolates of *A. xylosoxidans* were collected from two patients followed at Rigshospitalet in Copenhagen, Denmark. The use of the stored bacterial isolates was approved by the local ethics committee at the Capital Region of Denmark (Region Hovedstaden) with registration number H-4-2015-FSP. Two isolates were longitudinally collected from each patient: in 2005 and 2008 from patient A, and in 2008 and 2014 from patient B. Susceptibility to the following antibiotics was tested: amoxicillin + clavulanic acid, ampicillin, aztreonam, ceftazidime, ceftriaxone, cefuroxime, chloramphenicol, ciprofloxacin, colistin, imipenem, meropenem, moxifloxacin, penicillin, piperacillin + tazobactam, sulfamethoxazole, sulfamethoxazole + trimethoprim, tetracycline, tobramycin, trimethroprim. 

### 4.2. Library Preparation and Whole-Genome Sequencing

DNA was purified from over-night liquid cultures of single colonies using the DNEasy Blood and Tissue Kit (Qiagen). Libraries were made with Nextera XT and sequenced on an Illumina MiSeq using the v2 250 × 2 kit [20]. This project has been deposited at EMBL under the accession PRJEB35058. Sequence data can be found with the experiment accession numbers ERX3614542 (strain A1), ERX3614543 (strain A2), ERX3614548 (strain B1), and ERX3614549 (strain B2). The pair end sequencing yielded 3,842,110 reads for isolate A1; 2,947,162 reads for isolate A2; 2,806,776 reads for isolate B1; and 2,847,706 reads for isolate B2. As a reference genome sequence, the annotated genome *A. xylosoxidans* NH44784-1996 was used, which belongs to a strain isolated from sputum of a CF patient followed at Copenhagen CF Center in 1996 [10].

### 4.3. De Novo Assembly

The quality of the raw reads was assessed using FastQC v0.11.7, and adapter and quality trimming was performed accordingly following the illuminaclip, leading, trailing, slidingwindow and milen steps of Trimmomatic v0.36 [21] (Illuminaclip:adapter_file.fa:2:30:20 leading:3 trailing:3 slidingwindow:4:20 minlen:50). Sequence reads from each isolate were de novo assembled using the SPAdes v3.11.1 [22] assembly toolkit using the careful option. The quality of the de novo assemblies was evaluated using QUAST QC v5.0.0 [23] and by mapping the reads on the corresponding assembly using Bowtie 2 v2.3.4.1 [24] (coverage range: 97.87–98.35%); Samtools v1.9 [25] was used to obtain sorted bam files; Bedtools v2.27.1 [26] was used to obtain the per base sequence coverage; and Qualimap v2.2.1 [27] was used to obtain mapping and coverage statistics. All the de novo assemblies were annotated using Prokka v1.13 [28]. Genotypic relatedness was verified by checking core genome similarities obtained using the Harvest-OSX64-v1.1.2 suite [29].

### 4.4. Variant Analysis

Two types of variant analysis were carried out by aligning sequence reads to the *A. xylosoxidans* NH44784-1996 reference genome and to the de novo assembly of the longitudinal isolate from the same patient (reads from isolate 1 were aligned to the de novo assembly of isolate 2 from the same patient and vice versa) using Bowtie 2 v2.3.4.1. Sorted bam files were obtained using Samtools v1.9 and the MarkDuplicates tool from Picard v2.17.10 was used to mark duplicates. Finally, HaplotypeCaller of the Genome Analysis Toolkit (GATK) v4.0.6.0 [30] was used with the sample-ploidy option set to 1 to call SNPs and indels. In order to annotate the variants and predict their functional effects, SnpEff v4.3t [31] toolbox was used employing custom-built SnpEff databases obtained starting from fasta sequences and gff annotations of the genomes. Only variants supported by a minimum of 20 reads were retained.

For the analysis performed between the isolates, an ulterior step was performed in order to consider only true variants and discard false positives. Starting from the vcf files, the variants’ positions were extracted, and a bed file was created in order to extract from the de novo assembly the sequences containing the variant and a flanking region of 75 bp in each direction using Bedtools v2.27.1, thus obtaining 151 bp sequences. These were then mapped on the de novo assembly of the corresponding longitudinal isolate using Bowtie 2 v2.3.4.1 with the option end-to-end and setting the mismatch penalty to 0. Moreover, only the reads that mapped where a variant was called in the longitudinal isolate were kept. Finally, the occurrences of transitions and transversions were counted and the transitions/transversions ratio was calculated.

### 4.5. Mutator Genes Analysis

The genetic basis of hypermutation was investigated from whole genome sequencing data by analysing genes involved in this phenomenon [32]. The following genes present in the reference NH44784-1996 were considered: RS17415/*PfpI*, RS20390/*PfpI*, RS30630/*MutS*, RS06175/*MutL*, RS27975/*RadA*, RS21930/*Rad50*, RS11590/*UvrA*, RS27010/*UvrB*, RS31300/*UvrC*, RS27580/*UvrD*, and RS09700/*UvrD*.

### 4.6. Mobilome Analysis

The Phage Search Tool Enhanced Release (PHASTER) [33] was used in order to identify and annotate prophage sequences. The presence of plasmids was investigated in two ways. First, PlasmidFinder v2.0 [34] was used on the de novo assembled genomes. Moreover, the plasmidSPAdes pipeline was used on the whole genome sequencing dataset, and the DNA sequences of the putative plasmids were then blasted against the non-redundant (nr) BLAST database [35]. The presence of integrative and conjugative elements (ICEs), integrative and mobilizable elements (IMEs), and *cis*-mobilizable elements (CIMEs) was studied using the ICEfinder tool based on the ICEberg 2.0 database of bacterial integrative and conjugative elements [36]. 

### 4.7. Growth Curves

Bacterial strains were plated on LB agar and incubated at 37 °C for 24–48 h. One to two colonies were inoculated in 10 mL LB medium shaking at 37 °C overnight. OD_600_ was measured using a spectrophotometer, cultures were diluted to 0.05 OD/mL in LB medium and 150 µL/well were incubated in a 96-well plate for 20–24 h shaking at 37 °C. Using an automated plate reader, A_600_ was measured every 20 min. Growth rate was calculated using GraphPad Prism software.

### 4.8. Adhesion Assay

Bacterial strains were plated on LB agar and incubated at 37 °C for 24–48 h. One to two colonies were inoculated in 10 mL LB medium shaking at 37 °C overnight. OD_600_ was measured using a spectrophotometer, cultures were diluted to 0.05 OD/mL in LB medium and 150 µL/well were incubated in a 96-well plate for 20–24 h at 37 °C. After measuring A_600_, wells were washed twice with water to remove unattached cells, and surface-attached cells were stained with 0.1% crystal violet solution for 15 min. Wells were rinsed and washed three times with water, then dried for 1–2 h. Thirty percent acetic acid was added, incubated at room temperature for 15 min, and A_590_ was measured. Thirty percent acetic acid was used as blank. 

### 4.9. Protease Activity Measurement

*A. xylosoxidans* strains were plated on LB agar and incubated at 37 °C for 24–48 h. One to two colonies were inoculated in 10 mL LB medium shaking at 37 °C for 16 h. OD_600_ was measured and cultures were diluted to 0.1 OD/mL in 10 mL of LB medium. After shaking at 37 °C for 16 h, cultures were diluted to 1 OD/mL and centrifuged at 7000× *g* for 30 min at 4 °C. Supernatants were collected and sterile-filtered. Protease activity in culture supernatants was determined by azocasein assay as previously described [37]. Briefly, 350 µL reaction mixture containing 0.1 M Tris-HCl, pH 8.0, and 1% azocasein (Sigma-Aldrich, previously resuspended in 0.5% NaHCO_3_) was added to 150 µL supernatant and incubated at 37 °C for 20 min shaking. After the addition of 1 mL 7% ice-cold perchloric acid, the solution was centrifuged. One-hundred-and-fifty microlitres of 10 N sodium hydroxide were added to the clear supernatant and OD_430_ was measured. One protease unit was calculated as the amount of enzyme producing an increase of 0.1 OD per hour.

## Figures and Tables

**Table 1:** Information on the de novo assembly of each genome. Longitudinal isolates are numbered (1, 2) following the time of isolation.

Patient	Isolate	Genome Size (bp)	GC-Content (%)	No. Contigs	N50	Mean Coverage Depth (x)	No. Coding Sequences	Mapping Reads (%)
A	A1	6913734	68.09	291	78688	66	6359	98.3
A	A2	6879357	68.08	187	78799	50	6339	97.87
B	B1	6634994	67.63	178	100359	49	6041	98.07
B	B2	6628209	67.63	158	93753	40	6050	98.35

**Table 2:** Percentage of reads of each isolate mapping against the de novo assembly of the corresponding longitudinal isolate’s genome and against NH44784-1996 reference genome.

Isolate Reads	Longitudinal Isolate de novo Assembly	Mapping Reads vs de novo Assembly (%)	Mapping Reads vs NH44784-1996 (%)
A1	A2	96.49	52.37
A2	A1	97.31	46.27
B1	B2	98.78	83.08
B2	B1	96.73	82.98

**Table 3:** Genetic variants found in each genome, by type (SNPs, indel—transitions, transversions), by translational changes (synonymous, missense, nonsense, other) and by the predicted functional impact (frameshift, disruptive in-frame insertion/deletion, stop gain/loss).

Analysis	Comparison between Longitudinal Isolates		Comparison with Reference Genome			
Genome	A	B	A1	A2	B1	B2
Total	187	8	162	70	10	42
No. SNPs	150	6	150	68	8	39
No. indel	37	2	12	2	2	3
No. Synonymous SNPs	38	3	87	43	4	24
No. Missense SNPs	89	3	53	14	4	10
No. Nonsense SNPs	5	0	0	0	0	0
No. Other SNPs	18	0	10	11	0	5
Frameshift	13	2	8	0	0	2
Disruptive in-frame insertion	1	0	0	0	0	0
Disruptive in-frame deletion	0	0	0	0	0	1
Stop gain	5	0	1	0	0	0
Stop lost	1	0	0	0	0	0
Transitions	128	2	110	43	5	24
Transversions	22	4	40	25	3	15
Transition/transversion ratio	5.8	0.5	2.75	1.72	1.66	1.6

**Table 4:** List of gene-presenting variants, grouped by functional class. “Other” includes membrane protein, AsmA family protein, Tol-Pal system protein TolB.

Analysis	Comparison between Longitudinal Isolates			Comparison with Reference Genome				
Functional Category	A	B	Total	A1	A2	B1	B2	Total
Metabolism	66	0	66	49	18	3	4	74
Transcription and translation	21	1	22	13	3	1	4	21
Virulence, disease and defence	5	0	5	2	4	0	0	6
Hypothetical protein	34	2	36	4	1	2	3	10
Transporter	21	3	24	18	6	0	3	27
Iron acquisition and metabolism	10	0	10	6	1	0	1	8
Stress response	2	0	2	1	1	0	0	2
DNA repair	2	0	2	0	3	0	0	3
Antibiotic resistance	7	0	7	5	3	0	0	8
Mobile genetic elements	0	0	0	0	0	0	0	0
Other	3	0	3	4	0	0	0	4
Total	171	6	177	102	40	6	15	163

**Table 5:** Intact prophage regions, ICEs and IMEs found in each genome. Length of the regions is indicated in Kbp.

Mobile Elements	A1	A2	B1	B2
PHAGE_Burkho_KS9_NC_013055	21	21	-	-
PHAGE_Burkho_Bcep176_NC_007497	46.7	39	18.6	24.6
PHAGE_Salmon_118970_sal3_NC_031940	-	31.6	-	-
PHAGE_Pseudo_YMC11/02/R656_NC_028657	-	29.3	-	-
PHAGE_Burkho_BcepMu_NC_005882	-	-	40.2	40.9
PHAGE_Burkho_KS14_NC_015273	-	-	31.7	-
PHAGE_Aeromo_vB_AsaM_56_NC_019527	-	-	-	-
PHAGE_Synech_S_CBS1_NC_016164	-	-	-	-
ICEs	93	227	-	-
IMEs	-	-	15.6	15.6

## Supplementary Materials

The following are available online at https://www.mdpi.com/2076-0817/9/2/72/s1, Table S1: List of gene products affected by loss of function mutations in each genome versus the genome of its longitudinal isolate; Table S2: Presence of genes involved in DNA repair in each clinical isolate and in the reference strain.
