# Supplementary material for: Hypermutation as an Evolutionary Mechanism for Achromobacter xylosoxidans in Cystic Fibrosis Lung Infection
Source: Pathogens. 2020 Jan 21;9(2):72. doi: 10.3390/pathogens9020072 (PMC7168687; doi:10.3390/pathogens9020072)
Supplement: Supplementary file 1 [file pathogens-09-00072-s001.pdf]

Supplementary files

Table S1. List of gene products affected by loss of function mutations in each genome versus the genome of its longitudinal isolate. For hypothetical proteins, significant BLAST search suggestions (id>95%) are reported.

Genome	Variant type	Impact	Gene product
A	Frameshift variant	High	ABC transporter permease
A	Frameshift variant	High	Bifunctional diguanylate cyclase/phosphodiesterase
A	Frameshift variant	High	Hypothetical protein
A	Frameshift variant	High	16S rRNA (uracil(1498)-N(3))-methyltransferase
A	Frameshift variant	High	ABC transporter permease
A	Frameshift variant	High	Hypothetical protein (filamentous hemagglutinin N-terminal domain-containing protein - 99.94%)
A	Frameshift variant	High	Type II secretions system protein GspE
A	Frameshift variant	High	Leucyl aminopeptidase
A	Frameshift variant	High	Hypothetical protein (DUF4880 domain-containing protein - 100%)
A	Disruptive in-frame insertion	Moderate	Cytochrome ubiquinol oxidase subunit I
A	Stop gained	High	ABC transporter ATP-binding protein/permease
A	Stop gained	High	Sigma-70 family RNA polymerase sigma factor
A	Stop gained	High	exoU
A	Stop gained	High	Hypothetical protein

A	Stop gained	High	Putative 2-aminoethylphosphonate ABC transporter permease subunit
A	Stop lost	High	HlyD family efflux transporter periplasmic adaptor subunit
B	Frameshift variant	High	Efflux transporter outer membrane subunit
B	Frameshift variant	High	Hypothetical protein

Table S2. Presence of genes involved in DNA repair in each clinical isolate and in the reference strain.

Gene	NH44784_1996	A1	A2	B1	B2
pfp	2	2	2	2	2
mutS	1	1	1	1	1
mutL	1	2	2	2	2
sodA	1	0	0	0	0
sodB	1	0	0	0	0
sodC	1	0	0	0	0
radA	1	1	1	1	1
radC	1	0	0	0	0
rad50	1	1	1	1	1
uvrA	2	1	1	1	1
uvrB	2	1	1	1	1
uvrC	1	1	1	1	1
uvrD	2	2	2	3	3
